# Outcomes of Transobturator Tape Surgery for Stress and Mixed Urinary Incontinence: A 12-Year Retrospective Review

**DOI:** 10.3390/diagnostics15202611

**Published:** 2025-10-16

**Authors:** Cheng-Feng Lin, Hung-Yi Chen, Chun-Te Wu, Kuan-Lin Liu, Cheng-Chia Lin, Heng-Jung Hsu, Chin-Chan Lee, Chun-Yu Chen

**Affiliations:** 1 Department of Urology, Chang Gung Memorial Hospital, Keelung 204, Taiwan; b8801052@cgmh.org.tw (C.-F.L.); hongyi@cgmh.org.tw (H.-Y.C.); wucgmh@gmail.com (C.-T.W.); kuanlin@cgmh.org.tw (K.-L.L.); a97026@cgmh.org.tw (C.-C.L.); 2 College of Medicine, Chang Gung University, Taoyuan 333, Taiwan; hsuaaron@gmail.com (H.-J.H.); leefang@cgmh.org.tw (C.-C.L.); 3 Department of Nephrology, Chang Gung Memorial Hospital, Keelung 204, Taiwan

**Keywords:** transobturator tape, stress urinary incontinence, mixed urinary incontinence, overactive bladder symptom score, pelvic organ prolapse quantification, urodynamic predictors

## Abstract

**Background**: Stress urinary incontinence (SUI) and mixed urinary incontinence (MUI) are common disorders that impair quality of life. While transobturator tension-free vaginal tape (TVT-O) is established for SUI, outcomes in MUI remain uncertain. **Methods**: We analyzed 111 women who underwent TVT-O at Keelung Chang Gung Memorial Hospital. Baseline data included demographics, Overactive Bladder Symptom Score (OABSS), uroflowmetry [peak flow rate (PFR), residual urine (RU)], and Pelvic Organ Prolapse Quantification (POP-Q) stage. The primary outcome was OABSS improvement (≥1-point reduction); secondary outcomes were longitudinal OABSS, uroflowmetry, pad usage, and POP-Q stage. **Results**: At 3 months, 31.5% (35/111) met responder criteria. Symptom improvement occurred more often in MUI than in SUI, with about half of women with MUI (48.6%) and one quarter of those with SUI (27.4%) reporting subjective improvement (*p* = 0.018). OABSS improved in MUI (7.92 → 7.18, *p* = 0.001) but worsened in SUI (6.84 → 7.52, *p* < 0.001). In SUI, PFR increased (*p* = 0.001) and RU decreased (*p* = 0.029); no significant changes occurred in MUI. MUI independently predicted response (OR, 2.59; 95% CI, 1.10–6.14) and greater ΔOABSS (β = −1.391, *p* < 0.001); higher baseline OABSS also predicted improvement (β = −0.093, *p* = 0.049). For pad usage, MUI was associated with persistence (OR, 3.855, *p* = 0.010). ROC analysis showed modest discrimination for MUI (AUC 0.626, *p* = 0.034). **Conclusions**: TVT-O provided symptom relief, with about half of the women with MUI, and one quarter of those with SUI experienced subjective improvement. Women with MUI and higher baseline OABSS were more likely to improve, but these findings should be interpreted with caution, given the modest sample size.

## 1. Introduction

Stress urinary incontinence (SUI) is one of the most common pelvic floor disorders in women, with a lifetime risk of 30–40%, and substantially impairs quality of life [1]. When conservative therapy fails, mid-urethral sling (MUS) procedures are the gold-standard surgical treatment, offering high cure rates (62–98%) with minimal invasiveness [2]. Nonetheless, complications may occur, including de novo overactive bladder (OAB) symptoms in 6–20% of patients, as well as voiding dysfunction and urinary retention [3,4].

Several preoperative factors have been proposed as predictors of adverse outcomes, such as detrusor overactivity, reduced voided volume, impaired bladder capacity, and abdominal straining after MUS [3,4,5]. Patient-related variables, including age, menopausal status, and parity, may also influence efficacy and complication risk, yet the evidence remains inconsistent [3,4]. Mixed urinary incontinence (MUI) presents particular challenges, as surgical outcomes are generally less favorable than in pure SUI due to combined urethral deficiency and detrusor overactivity [6]. While pharmacologic agents, local estrogen, and intradetrusor botulinum toxin can relieve refractory postoperative OAB [7,8], current evidence is limited regarding which preoperative clinical and urodynamic factors reliably predict surgical outcomes, especially in women with MUI or functional bladder abnormalities [7,8].

The tension-free vaginal tape (TVT) and transobturator tape (TVT-O) procedures are standard surgical treatments for women with SUI. Although these MUSs are associated with high rates of long-term success, outcomes vary among patient subgroups [9,10]. The efficacy of TVT-O for treating MUI in women, particularly those with a predominance of stress-related symptoms, has been well-established through multiple randomized controlled trials and long-term follow-up studies. TVT-O demonstrates good subjective and objective success rates and a significant improvement in quality of life. Long-term follow-up studies (3–9 years) have shown subjective success rates of 65–74%, with some patients also experiencing relief from their urgency symptoms [11,12,13]. However, the treatment effect of TVT-O is relatively limited for women with MUI where urgency is the dominant symptom. In some cases, urgency symptoms may persist or even worsen after the surgery. Therefore, a clear preoperative assessment of the patient’s dominant symptom type is crucial. Guidelines from the American Urological Association (AUA) and the Society of Urodynamics, Female Pelvic Medicine, and Urogenital Reconstruction (SUFU) indicate that both TVT-O and TVT are standard surgical options for women with stress-predominant MUI. In contrast, for those with urgency-predominant symptoms, conservative or medical management should be the first-line consideration [14].

In conclusion, the effectiveness of TVT-O for urgency-predominant MUI remains limited, and treatment should be individualized. The predictive value of baseline clinical and urodynamic parameters is still uncertain, highlighting the need to identify reliable prognostic factors to optimize patient selection and counseling. Therefore, we conducted this study to evaluate the efficacy of TVT-O in women with SUI or MUI and to examine baseline demographic, clinical, pad usage, and urodynamic factors, with particular focus on the prognostic roles of MUI and pelvic organ prolapse (POP) stage.

## 2. Materials and Methods

### 2.1. Study Design and Population

We performed a retrospective review of women who underwent TVT-O surgery at Chang Gung Memorial Hospital, Keelung Branch, between January 2012 and May 2024. Clinical information was extracted from medical records, including demographic characteristics, medical and surgical history, incontinence features (e.g., symptom duration and pad usage), pre- and postoperative urodynamic studies, Overactive Bladder Symptom Score (OABSS), Pelvic Organ Prolapse Quantification (POP-Q) stage, and surgical complications. The OABSS was obtained using a patient-completed questionnaire, with clinical staff available to clarify items if needed. POP-Q staging was performed by attending urogynecologic surgeons during the preoperative pelvic examination.

**Figure 1:**
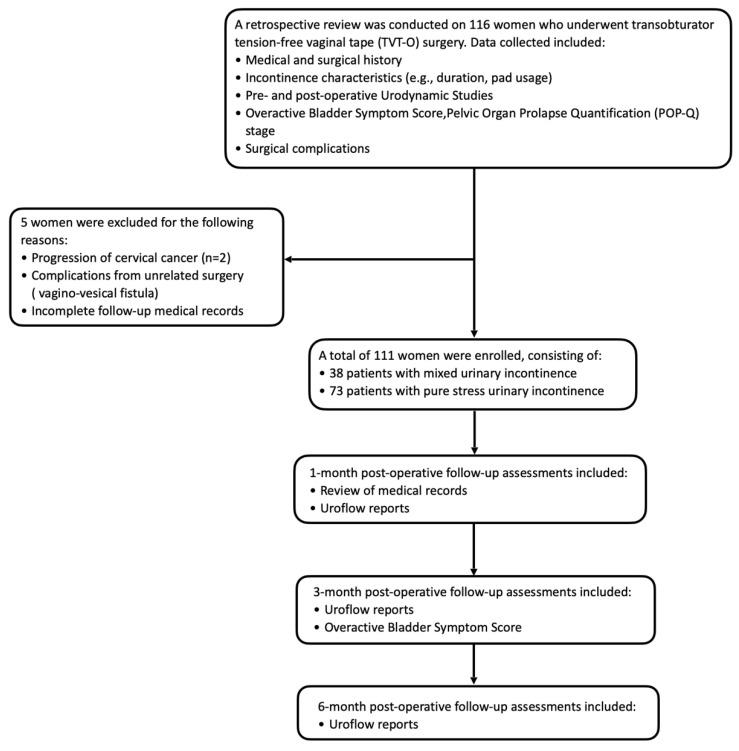
Flowchart of study subjects’ enrollment.

A total of 116 women were initially screened for inclusion in this study. Subjects were excluded if they had progression of cervical cancer (*n* = 2), complications from unrelated pelvic surgery (e.g., vesicovaginal fistula), or incomplete follow-up records (*n* = 3). After these exclusions, 111 women remained eligible for analysis, comprising 38 participants with MUI and 73 with pure SUI (Figure 1). All procedures were performed at the Keelung Branch of Chang Gung Memorial Hospital, a district general hospital in northern Taiwan. The center performs approximately 30–40 mid-urethral sling procedures annually, although case numbers temporarily declined during the COVID-19 pandemic (2019–2022).

MUI was defined as the coexistence of stress urinary incontinence and urgency urinary incontinence, as documented in clinical history and confirmed by urodynamic testing. SUI was defined as involuntary urine leakage during increased intra-abdominal pressure (e.g., coughing or exertion) in the absence of urgency symptoms.

All women had previously attempted conservative management, including pelvic floor training and/or pharmacologic therapy, before surgical intervention was considered. Eligibility for TVT-O required persistent SUI or MUI confirmed by clinical and urodynamic evaluation. Each patient participated in a shared decision-making (SDM) process, received detailed counseling regarding the risks, benefits, and alternatives to surgery, and signed a written informed consent form for the surgical procedure after being given at least one week to consider the decision.

The study was conducted in accordance with the principles of the Declaration of Helsinki. The study protocol was reviewed and approved by the Institutional Review Board of the Chang Gung Medical Foundation (approval number: 202500883B0). The requirement for a separate research-specific informed consent was waived because of the retrospective design and use of de-identified data.

### 2.2. Follow-Up and Assessments

Postoperative outcomes were evaluated at 1, 3, and 6 months after surgery. At the 1-month visit, follow-up included review of medical records and uroflowmetry. At 3 months, participants underwent uroflowmetry and assessment with the OABSS. At 6 months, uroflowmetry was repeated. These assessments provided objective measures of voiding function, symptom severity, and anatomic outcomes over time.

### 2.3. Procedural Details of TVT-O Surgery

All TVT-O procedures were performed by senior urogynecologic surgeons following a standardized technique. Surgery was carried out under general anesthesia with the patient placed in the dorsal lithotomy position, with hips hyperflexed and buttocks aligned with the edge of the operating table; the head was tilted approximately 15 degrees downward. A midline vaginal incision was made at the level of the mid-urethra, and bilateral paraurethral tunnels were created toward the obturator foramen. The polypropylene tape was introduced through the obturator foramen using helical trocars, either by the inside-out or outside-in technique, and positioned beneath the mid-urethra without tension.

Intraoperative cystoscopy was routinely performed to exclude bladder or urethral injury. Hemostasis was secured, and the vaginal incision was closed with absorbable sutures. A Foley catheter was left in place and usually removed within 24 h after surgery. Short-term perioperative complications were monitored but were not included in the present analysis, as the primary focus of this study was on long-term functional outcomes assessed by the OABSS.

### 2.4. Outcomes Measures

The primary outcome was improvement in overactive bladder symptoms, defined as a reduction of at least 1 point in the OABSS (ΔOABSS ≤ −1) from baseline to postoperative follow-up.

Secondary outcomes included (1) longitudinal changes in OABSS across 1-, 3-, and 6-month follow-up visits, analyzed with repeated-measures ANOVA; (2) uroflowmetry parameters, including peak flow rate (PFR, defined as the maximum urinary flow rate during voiding) and postvoid residual urine (RU) volume; (3) postoperative pad usage; and (3) anatomical findings at baseline, assessed by the POP-Q system. Subgroup analyses compared outcomes between women with MUI and those with SUI.

### 2.5. Statistical Analysis

Continuous variables are presented as means ± standard deviations or medians with interquartile ranges, as appropriate, and categorical variables as frequencies and percentages. Baseline characteristics between women with MUI and those with SUI were compared with Student’s *t*-test or the chi-square test.

Longitudinal changes in the OABSS over 1-, 3-, and 6-month follow-up visits were analyzed with repeated-measures analysis of variance (ANOVA). Linear regression models were used to examine associations between baseline clinical variables and changes in OABSS. Logistic regression was performed to identify predictors of treatment response, defined as a reduction of at least 1 point in OABSS. Odds ratios (ORs) with 95% confidence intervals (CIs) were reported.

Receiver-operating characteristic (ROC) curve analysis was conducted to assess the ability of OABSS changes to discriminate treatment response.

All statistical analyses were conducted using the Statistical Package for the Social Sciences (SPSS, version 27.0.1.0 for Mac; IBM Corp., Armonk, NY, USA), and graphs were generated with GraphPad Prism, version 10; GraphPad Software, San Diego, CA, USA). A two-sided *p* value < 0.05 was considered statistically significant.

## 3. Results

### 3.1. Study Design and Subject Characteristics

A total of 111 women were included in the final analysis, comprising 38 with MUI and 73 with SUI. The mean age of the study population was 60.5 ± 10.9 years. Baseline characteristics were generally comparable between the two groups. Women with MUI tended to be slightly older than those with SUI (62.9 ± 10.0 vs. 59.3 ± 10.7 years, *p* = 0.091), although the difference was not statistically significant. The prevalence of comorbidities, including diabetes, chronic kidney disease, heart failure, and prior hysterectomy, did not differ significantly between groups.

The number of pads used before surgery was similar in both groups. After surgery, 14 of 38 women with MUI (36.8%) still required pads, whereas only 10 of 73 women with SUI (13.9%) did so (*p* = 0.006). Other clinical parameters, including body mass index, severity of bladder prolapse (POP-Q stage), duration of incontinence before surgery, use of anti-incontinence medications, detrusor contraction pressure, and maximum urethral closure pressure, showed no significant differences between the groups (Table 1).

### 3.2. Changes in Symptom Scores and Urodynamic Parameters

During follow-up, OABSS improved significantly over time in both groups (*p* for time < 0.001). The mean OABSS decreased from 6.84 ± 3.62 at baseline to 7.52 ± 3.91 at 3 months in the SUI group, and from 7.92 ± 4.04 to 7.18 ± 3.70 in the MUI group (*p* for time = 0.001). No significant interaction was observed between group and time, indicating that the magnitude of improvement was comparable between MUI and SUI (Table 2).

Regarding uroflowmetry, PFR increased significantly over time in the SUI group (*p* = 0.001), whereas changes in the MUI group were not significant. Residual urine volume decreased significantly only in the SUI group (*p* = 0.029), while voiding volume showed no consistent differences over time in either group.

**Figure 2:**
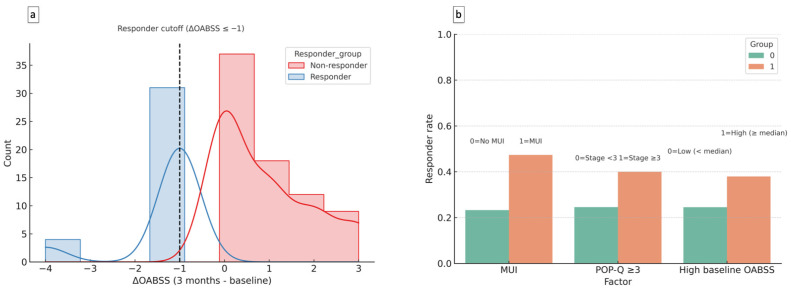
Distribution of treatment response and subgroup analysis. Panel (**a**): Distribution of ΔOABSS at 3 months compared with baseline, stratified by responder status. Responders were defined as ΔOABSS ≤ −1 (blue), and non-responders as ΔOABSS > −1 (red). The dashed vertical line indicates the responder cutoff (ΔOABSS ≤ −1). Panel (**b**): Subgroup responder rates according to baseline characteristics, including MUI, POP-Q stage ≥ 3, and baseline OABSS (high vs. low, dichotomized at the median). Bars represent the proportion of responders within each subgroup. Abbreviations: MUI, mixed urinary incontinence; OABSS, Overactive Bladder Symptom Score; POP-Q, Pelvic Organ Prolapse Quantification.

Based on the predefined responder definition (ΔOABSS ≤ −1), 48.6% of MUI subjects and 27.4% of SUI subjects were classified as responders (Figure 2a). Subgroup analyses showed higher responder rates among women with MUI, those with POP-Q stage ≥ 3, and those with higher baseline OABSS (Figure 2b).

These results indicate that TVT-O surgery was associated with significant symptom relief and improvements in voiding function, particularly in women with SUI, while women with MUI derived relatively greater subjective benefit.

### 3.3. Predictors of Postoperative Pad Usage

Univariate analysis showed that parity (OR, 1.552; 95% CI, 1.014–2.376; *p* = 0.043) and MUI (OR, 3.617; 95% CI, 1.415–9.244; *p* = 0.007) were significantly associated with postoperative pad usage (Table 3). In multivariable logistic regression, MUI remained an independent predictor of postoperative pad usage in both adjusted models. In Model 1 (adjusted for MUI, parity, and residual urine), MUI was associated with 3.57-fold higher odds of pad usage (95% CI, 1.357–9.393; *p* = 0.010). In Model 2 (adjusted for MUI, parity, residual urine, peak flow rate, BMI, and age), MUI remained significant (OR, 3.855; 95% CI, 1.379–10.778; *p* = 0.010). These findings indicate that participants with MUI were more likely to require pad use after TVT-O surgery, independent of other baseline clinical factors.

### 3.4. Baseline Factors Associated with Symptom Response

Among the 111 participants, 35 (31.5%) were classified as responders (ΔOABSS ≤ −1) and 76 (68.5%) as non-responders (Table 4). Responders tended to be older (62.9 ± 10.7 vs. 59.4 ± 10.8 years), although the difference was not statistically significant (*p* = 0.109). The prevalence of diabetes, heart failure, and advanced POP-Q stage did not differ significantly between the two groups.

Baseline OABSS was numerically higher among responders than non-responders (8.17 ± 3.75 vs. 6.76 ± 3.75), although the difference did not reach statistical significance (*p* = 0.070). MUI was significantly more common in responders than in non-responders (51.4% vs. 26.3%, *p* = 0.018). Other baseline urodynamic parameters, including residual urine volume, voiding volume, and peak flow rate, were not significantly associated with response status.

In the MUI group, 18 of 38 women (47.4%, 95% CI, 30.9–64.2) were classified as responders, compared with 20 of 73 women (27.4%, 95% CI, 17.6–39.1) in the SUI group. Although women with MUI reported relatively greater subjective improvement, their objective outcomes, such as postoperative pad usage, were less favorable. These subgroup findings should be interpreted as exploratory, given the limited sample size.

**Figure 3:**
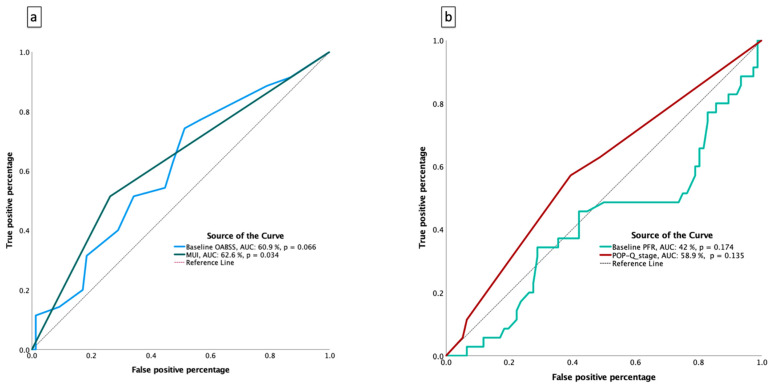
Receiver-operating characteristic curves for predicting treatment response. ROC curve analysis was performed to evaluate baseline factors associated with treatment response, defined as a reduction of at least 1 point in OABSS. Panel (**a**): ROC curves for MUI, baseline OABSS, PFR, and POP-Q stage ≥ 3. Panel (**b**): Comparative plots showing the discriminatory ability of these baseline variables. Among them, MUI showed a significant association with treatment response (AUC 0.626, *p* = 0.034), whereas baseline OABSS, PFR, and POP-Q stage ≥ 3 were not statistically significant predictors. Abbreviations: ROC, receiver-operating characteristic; OABSS, Overactive Bladder Symptom Score; MUI, mixed urinary incontinence; PFR, peak flow rate; POP-Q, Pelvic Organ Prolapse Quantification.

Receiver-operating characteristic (ROC) analysis showed that MUI was a significant factor associated with treatment response (AUC 0.626, *p* = 0.034) (Figure 3a). In contrast, baseline OABSS (AUC 0.609, *p* = 0.066), baseline PFR (AUC 0.420, *p* = 0.174), and POP-Q stage ≥ 3 (AUC 0.589, *p* = 0.135) were not statistically significant predictors of response (Figure 3a,b). Overall, MUI at baseline emerged as the most important factor associated with symptomatic improvement following TVT-O surgery.

### 3.5. Multivariable Analysis of Treatment Response

In simple linear regression, MUI was significantly associated with greater improvement in OABSS at 3 months (β = −1.422 ± 0.262, *p* < 0.001), whereas diabetes also showed an association with symptom change (β = 0.887 ± 0.341, *p* = 0.011) (Table 5). In multiple regression, MUI remained an independent factor associated with OABSS improvement in both Model 1 (β = −1.377 ± 0.264, *p* < 0.001) and Model 2 (β = −1.391 ± 0.275, *p* < 0.001). POP-Q stage ≥ 3 was also significantly associated with OABSS change in Model 2 (β = −0.093 ± 0.047, *p* = 0.049).

**Figure 4:**
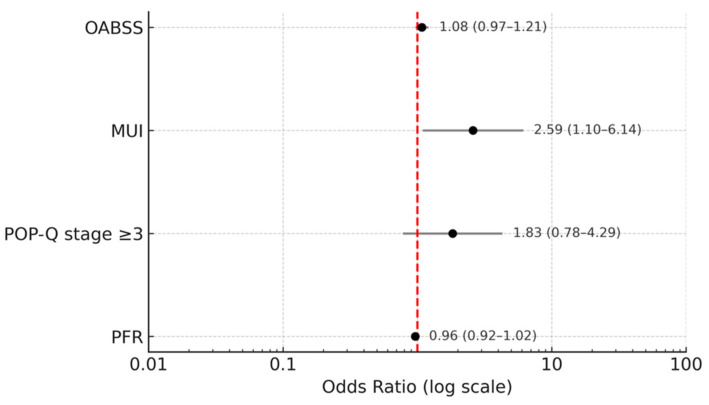
Multivariable logistic regression for factors associated with treatment response. Forest plot displaying odds ratios with 95% confidence intervals from multivariable logistic regression analysis of factors associated with treatment response. The red dashed vertical line represents an odds ratio of 1.0 (the line of no effect). Responder status was defined as a reduction of at least 1 point in OABSS. Abbreviations: OABSS, Overactive Bladder Symptom Score; MUI, mixed urinary incontinence; PFR, peak flow rate; POP-Q, Pelvic Organ Prolapse Quantification.

Forest plot analysis of multivariable logistic regression (Figure 4) further demonstrated that MUI was significantly associated with treatment response (OR, 2.59; 95% CI, 1.10–6.14). In contrast, baseline OABSS, POP-Q stage ≥ 3, and PFR were not significant predictors of responder status.

Together, these analyses highlight MUI as the strongest baseline factor associated with symptomatic improvement after TVT-O surgery, with additional contribution from advanced POP-Q stage.

### 3.6. Perioperative and Postoperative Complications

Perioperative complications were rare (Table 6). Urinary retention occurred in 5 women (4.5%) and was successfully managed with temporary catheterization, without a need for further intervention. One woman (0.9%) developed a urinary tract infection, which resolved after oral antibiotic therapy. No cases of bladder perforation, hematoma, groin pain, or sling exposure were observed during the study period.

## 4. Discussion

In this study of 111 women who underwent TVT-O surgery, we observed significant improvements in overactive bladder symptoms as measured by the OABSS, together with selective gains in voiding function. Although postoperative increases in PFR and reductions in residual urine were more evident in women with SUI, women with MUI demonstrated a higher rate of symptom response. Baseline analysis consistently identified MUI as a key factor associated with postoperative improvement, whereas the POP-Q stage showed a possible association, and other clinical or urodynamic variables had limited predictive value. Taken together, these findings challenge the traditional view that MUI predicts poorer outcomes and underscore the importance of symptom subtype in surgical decision-making.

The efficacy of MUS procedures, including TVT-O, for the treatment of SUI has been well documented, with reported long-term success rates typically ranging from 70% to 90%, and most series report rates above 80% [15,16,17,18]. However, outcomes in women with MUI remain more controversial, with multiple studies demonstrating that cure rates are significantly lower and the persistence of urgency or urge urinary incontinence symptoms is higher compared with those with pure SUI. The presence of severe baseline urgency symptoms or detrusor overactivity also predicts poorer outcomes [19,20,21]. Our findings differ from these reports, showing that women with MUI achieved a higher rate of symptom response than those with SUI. This discrepancy may reflect differences in patient selection, baseline symptom burden, or definitions of treatment response across studies. Few prior investigations have directly examined the contribution of the POP-Q stage to postoperative symptom relief after MUS procedures. Our observation of a possible association adds to the limited and largely indirect evidence suggesting that pelvic support status may influence these outcomes [22,23].

Several mechanisms may account for the greater symptom improvement observed in women with MUI. Restoration of mid-urethral support not only reduces urethral hypermobility but may also diminish reflex detrusor contractions triggered by afferent nerves in the urethra during stress-related urine leakage, a phenomenon sometimes referred to as the “urethral reflex” [24,25]. This reflex likely contributes to the urgency symptoms seen in MUI patients [1,2]. By eliminating stress-induced urine loss, MUS procedures such as TVT-O can improve the stress incontinence component of MUI. Some patients also experience improvement in urgency symptoms, although the mechanism is not fully understood and may involve changes in afferent signaling. Additionally, we observed a postoperative increase in the average voided volume among MUI subjects in our study, suggesting improved bladder storage capacity, which may directly alleviate urgency and frequency, the key symptoms of OAB [3,26,27].

Psychosocial factors, including anxiety related to unpredictable leakage, can heighten urgency perception in women with MUI. Surgical control of stress incontinence with MUS sling procedures such as TVT-O is associated with significant improvements in anxiety, depression, and quality of life, which may contribute to the observed improvement in OAB symptoms in some patients. However, the relationship is multifactorial, and not all patients will experience resolution of urgency or OAB symptoms solely through reduction of psychological stress [4,28,29,30]. In contrast, among our study participants, women with pure SUI did not experience similar improvement in urgency symptoms; in fact, their OABSS tended to worsen. This phenomenon may be explained by the development of de novo urgency or OAB symptoms in some patients, potentially related to increased urethral resistance or altered bladder sensation after sling placement. Such changes could elevate detrusor pressure or sensory sensitivity and thereby precipitate new urgency, as supported by reports that a minority of women with SUI develop these symptoms after MUS procedures [5,31]. Collectively, these observations suggest that the pathophysiology of urgency in MUI is more complex and heterogeneous than in pure SUI. While surgical correction of urethral incompetence may relieve urgency symptoms in some patients, not all women experience parallel improvements, underscoring the need for individualized treatment strategies. Moreover, although our data do not demonstrate objective superiority of TVT-O in MUI compared with SUI, this modest real-world study provides encouraging evidence that many women with MUI still report meaningful subjective benefit. One possible explanation is that women with MUI had more severe baseline incontinence, so even partial physical improvement after surgery translated into substantial psychological satisfaction.

POP was present in approximately half of the participants in our cohort. POP typically manifests with voiding dysfunction and urinary retention rather than stress incontinence, whereas the indication for TVT-O in this study was SUI or MUI. Importantly, none of the women underwent concomitant prolapse repair at the time of sling surgery, thereby minimizing potential confounding from additional procedures. Although POP was systematically evaluated preoperatively using the POP-Q system, our analysis was not designed or powered to assess the independent impact of POP or prolapse repair on continence outcomes. This potential confounder warrants further clarification in future prospective studies.

The overall complication rate after TVT-O in our cohort was low. Transient urinary retention, the most common adverse event, occurred in 5 subjects (4.5%) and was successfully managed with short-term catheterization. One patient (0.9%) developed a urinary tract infection, which resolved with conservative therapy. Importantly, we observed no bladder perforation, hematoma, groin pain, or sling exposure during follow-up.

While these findings are reassuring, it is important to interpret them within the broader context of international concerns regarding mesh-related complications. The controversies highlighted by the Independent Medicines and Medical Devices Safety (IMMDS) Review primarily related to transvaginal mesh for POP repair, which carries higher risks of erosion and chronic pain [32]. In contrast, MUS procedures such as TVT and TVT-O, although constructed from similar polypropylene material, have consistently demonstrated a favorable safety profile and remain endorsed as the standard surgical treatment for SUI in international guidelines [33,34]. In the United Kingdom, the IMMDS Review led to a national pause on vaginal mesh procedures for stress urinary incontinence in 2018, later extended to prolapse repair, with the Government issuing its formal response in 2021 to strengthen safeguards and ensure high-vigilance practice. Importantly, the safety and efficacy of MUSs have been validated by multiple randomized trials and long-term cohort studies, which consistently report high cure rates and low complication rates compared with prolapse mesh procedures [18]. Our results are consistent with this distinction, but continued vigilance, careful patient selection, and comprehensive counseling remain essential to ensure patient safety. Moreover, long-term follow-up studies are warranted to further confirm the durability and safety of sling procedures.

These results provide several important clinical insights. Women with MUI should not be regarded as poor candidates for TVT-O, as many achieved meaningful symptom relief across both stress and urgency domains. Women with advanced POP-Q stage, reflecting more severe bladder prolapse, may also represent a subgroup that could particularly benefit from surgery, and this observation may encourage offering TVT-O to such women. At the same time, the heterogeneity of urgency outcomes underscores the importance of individualized preoperative counseling, with clear communication that not all women will experience parallel improvements in storage symptoms. It should also be noted that, although women with MUI reported relatively greater subjective improvement, their objective results, such as postoperative pad usage, were less favorable. This discrepancy indicates that patient-reported outcomes and objective measures may not always align, underscoring the exploratory nature of our subgroup findings. Taken together, these implications support a more refined approach to patient selection and counseling for women considering TVT-O.

In summary, this review of 111 women undergoing TVT-O surgery provides a straightforward message for clinical practice. About half of those with MUI and one in four with SUI experienced meaningful subjective improvement after surgery. Pad use decreased for many, although some women, especially those with MUI, still required pads. The procedure was safe, with only a few short-term complications.

This study has several limitations. It was conducted at a single center with a modest sample size, which may limit the generalizability of the findings. The retrospective design raises the possibility of selection bias and unmeasured confounding. In particular, our study was not powered to detect subgroup differences with high precision. Therefore, both the findings within the MUI subgroup and the comparative analyses between MUI and SUI should be regarded as exploratory and interpreted with caution. Follow-up was limited to 6 months, precluding conclusions regarding long-term durability of symptom improvement. Moreover, postoperative POP-Q assessment was not routinely performed, which prevented us from evaluating the postoperative course of prolapse and its potential influence on continence outcomes. Perioperative complications were not systematically analyzed, although most were transient and not expected to alter the main outcomes. In addition, OABSS was obtained through patient self-report, which may be subject to recall or reporting bias; however, this limitation is inherent to symptom-based instruments and was mitigated by the use of a validated questionnaire administered consistently across all participants. These limitations should be considered when interpreting the results.

## 5. Conclusions

TVT-O surgery may provide meaningful symptom relief in women with both SUI and MUI. Approximately half of women with MUI and one quarter of those with SUI reported subjective improvement after surgery. Higher baseline OABSS and the presence of MUI were associated with greater postoperative improvement, whereas objective urodynamic parameters were not predictive of outcomes. These findings highlight the relevance of patient-reported symptom measures in guiding treatment expectations and support a balanced, individualized approach to counseling. Further validation in larger, prospective, multicenter studies is warranted.

## Figures and Tables

**Table 1:** Baseline demographic and clinical characteristics of women with urinary incontinence treated with TVT-O.

	Overall (*n* = 111)	MUI Group (*n* = 38)	SUI Group (*n* = 73)	*p* Value
Age, years	60.50 ± 10.86	62.92 ± 10.99	59.25 ± 10.65	0.091
Diabetes, *n* (%)	22 (19.8)	5 (13.2)	17 (23.3)	0.204
Chronic kidney disease, *n* (%)	27 (24.3)	10 (26.3)	17 (23.3)	0.724
Pelvic radiotherapy, *n* (%)	3 (2.7)	1 (2.6)	2 (2.7)	1.000
Heart failure	9 (8.1)	4 (10.5)	5 (6.8)	0.501
Bladder trabeculation, *n* (%)	19 (17.3)	9 (24.3)	10 (13.7)	0.164
History of hysterectomy, *n* (%)	30 (27.0)	12 (31.6)	18 (24.7)	0.436
Parity, *n* (%)				0.537
1 delivery	10 (9.0)	4 (10.5)	6 (8.2)	
2 deliveries	42 (37.8)	13 (34.2)	29 (39.7)	
≥3 deliveries	59 (53.2)	21 (55.3)	38 (52.1)	
Body mass index, kg/m^2^	25.14 ± 4.04	24.63 ± 3.69	25.41 ± 4.21	0.346
Pad used before surgery, *n* (%)				0.784
0 pads/day	5 (4.5)	2 (5.4)	3 (4.1)	
1 pad/day	62 (56.4)	22 (59.5)	40 (54.8)	
≥2 pads/day	43 (39.1)	13 (35.1)	30 (41.1)	
Pad used after surgery, *n* (%)	24 (21.8)	14 (36.8)	10 (13.9)	0.006 *
Severity of bladder prolapse (POP-Q stage), *n* (%)				0.246
Stage < 3	61 (55.0)	18 (47.4)	43 (58.9)	
Stage ≥ 3	50 (45.0)	20 (52.6)	30 (41.1)	
Duration of urinary incontinence before surgery, *n* (%)				0.572
<12 months	37 (33.3)	14 (36.8)	23 (31.5)	
≥12 months	74 (66.7)	24 (63.2)	50 (68.5)	
Repeat TVT-O	6 (5.4)	2 (5.3)	4 (5.5)	0.962
Preoperative anti-incontinence medications, *n* (%)				0.366
0–1 medication	79 (71.2)	25 (65.8)	54 (74.0)	
≥2 medications	32 (28.8)	13 (34.2)	19 (26.0)	
Preoperative detrusor contraction pressure, cmH_2_O	48.36 ± 31.78	44.27 ± 31.97	50.43 ± 31.70	0.339
Preoperative MUCP, cmH_2_O	58.47 ± 19.47	56.13 ± 19.93	59.71 ± 19.23	0.362

Abbreviations: TVT-O, transobturator tension-free vaginal tape; SUI, stress urinary incontinence; MUI, mixed urinary incontinence; Parity, number of childbirths (live births); POP-Q, Pelvic Organ Prolapse Quantification; MUCP, maximum urethral closure pressure. Severity of bladder prolapse was categorized according to the Pelvic Organ Prolapse Quantification (POP-Q) system. *: *p* value < 0.05.

**Table 2:** Preoperative and postoperative urodynamic parameters and symptom scores in women undergoing TVT-O.

Variable	Group	Baseline (Pre-op)	1 Month	3 Months	6 Months	*p* (Time)	*p* (Group)	*p* (Interaction)
RU, mL	SUI	19.32 ± 22.90	18.94 ± 28.36	—	13.55 ± 26.97	0.029 *	0.727	0.020 * (1M), 0.624 (6M)
	MUI	21.84 ± 28.32	43.42 ± 76.96	—	20.68 ± 29.61	0.444		
PFR, mL/s	SUI	20.68 ± 29.61	20.53 ± 8.20	—	22.82 ± 8.15	0.001 *	0.051	ns
	MUI	22.05 ± 8.11	19.18 ± 11.89	—	19.97 ± 9.32	0.250		
Voiding volume, mL	SUI	299.70 ± 110.95	269.36 ± 128.24	—	283.80 ± 108.99	0.147	0.129	0.061 (1M), 0.097 (6M)
	MUI	283.97 ± 103.95	233.48 ± 126.93	—	293.99 ± 127.76	0.009 *		
OABSS	SUI	6.84 ± 3.62	—	7.52 ± 3.91	—	<0.001 *	0.233	ns
	MUI	7.92 ± 4.04	—	7.18 ± 3.70	—	0.001 *		

Abbreviations: TVT-O, transobturator tension-free vaginal tape; SUI, stress urinary incontinence; MUI, mixed urinary incontinence; RU, residual urine volume; PFR, peak flow rate; OABSS, Overactive Bladder Symptom Score. Values are presented as mean ± standard deviation. *p* (time): effect of time within subjects (repeated measures/linear mixed model or paired *t*-test for OABSS). *p* (group): effect of group (SUI vs. MUI); *p* (interaction): global interaction effect plus post hoc simple effects at each time point. ns = not significant; *: *p* value < 0.05.

**Table 3:** Univariate and multivariable logistic regression models identifying predictors of postoperative pad usage after TVT-O.

	Univariate, Crude			Multivariable, Model 1			Multivariable, Model 2		
	OR	95% CI	*p* Value	OR	95% CI	*p* Value	OR	95% CI	*p* Value
Age	1.025	0.983–1.069	0.249	—	—	—	1.004	0.956–1.056	0.864
BMI	1.070	0.958–1.195	0.233	—	—	—	1.100	0.967–1.250	0.149
Diabetes	1.578	0.536–4.641	0.407	—	—	—	—	—	—
Heart failure	1.905	0.439–8.258	0.389	—	—	—	—	—	—
Parity	1.552	1.014–2.376	0.043 *	1.454	0.919–2.300	0.110	1.478	0.906–2.411	0.118
Pre-op pad usage	1.098	0.795–1.515	0.571	—	—	—	—	—	—
POP-Q stage	0.925	0.700–1.222	0.583	—	—	—	—	—	—
Residual urine	1.014	0.998–1.031	0.086	1.011	0.993–1.029	0.228	1.008	0.990–1.026	0.379
Voiding volume	0.998	0.993–1.002	0.356	—	—	—	—	—	—
Peak flow rate	0.956	0.902–1.012	0.123	—	—	—	0.951	0.889–1.017	0.140
MUI	3.617	1.415–9.244	0.007 *	3.570	1.357–9.393	0.010 *	3.855	1.379–10.778	0.010 *

Abbreviations: TVT-O, transobturator tension-free vaginal tape; OR, odds ratio; CI, confidence interval; BMI, body mass index; Parity, number of childbirths (live births); POP-Q, Pelvic Organ Prolapse Quantification; MUI, mixed urinary incontinence. Model 1 adjusted for MUI, parity, and residual urine. Model 2 adjusted for MUI, parity, residual urine, peak flow rate, BMI, and age. *: *p* value < 0.05.

**Table 4:** Baseline characteristics between OABSS responders and non-responders.

	Responder (ΔOABSS ≤ −1, *n* = 35)	Non-Responder (ΔOABSS > −1, *n* = 76)	*p* Value
Age, years (mean ± SD)	62.94 ± 10.70	59.38 ± 10.81	0.109
BMI, kg/m^2^ (mean ± SD)	25.25 ± 3.73	25.09 ± 4.20	0.836
Parity, *n* (mean ± SD/median)	2.83 ± 1.10/3	2.57 ± 0.99/2	0.279
MUI, *n* (%)	18 (51.4)	20 (26.3%)	0.018 *
Baseline OABSS (mean ± SD)	8.17 ± 3.75	6.76 ± 3.75	0.070
Diabetes, *n* (%)	4 (11.4)	18 (23.7%)	0.212
Heart failure, *n* (%)	2 (5.7)	7 (9.2%)	0.800
POP-Q stage ≥ 3, *n* (%)	20 (57.1)	30 (39.5%)	0.125
Residual urine (mean ± SD)	25.83 ± 31.00	17.58 ± 21.07	0.159
Voiding volume (mean ± SD)	277.99 ± 127.72	301.83 ± 98.26	0.332
Peak flow rate (mean ± SD)	21.31 ± 8.29	24.33 ± 8.95	0.086
No. of anti-incontinence meds	1.09 ± 0.98	0.89 ± 0.83	0.322

Abbreviations: OABSS, Overactive Bladder Symptom Score; BMI, body mass index; MUI, mixed urinary incontinence; POP-Q, Pelvic Organ Prolapse Quantification. *: *p* value < 0.05.

**Table 5:** Linear regression of baseline factors for ΔOABSS at 3 months.

	Simple Linear Regression		Multiple Regression Analysis, Model 1		Multiple Regression Analysis, Model 2	
	β ± SE	*p* Value	β ± SE	*p* Value	β ± SE	*p* Value
Age	−0.009 ± 0.013	0.505	—	—	0.008 ± 0.013	0.509
BMI	0.015 ± 0.035	0.677	—	—	0.010 ± 0.033	0.756
Diabetes	0.887 ± 0.341	0.011 *	—	—	—	—
Heart failure	−0.578 ± 0.510	0.260	—	—	—	—
Parity	−0.159 ± 0.134	0.238	—	—	−0.108 ± 0.134	0.422
Baseline OABSS	−0.065 ± 0.037	0.079	−0.041 ± 0.033	0.216	−0.093 ± 0.047	0.049 *
POP-Q stage	−0.014 ± 0.085	0.868	—	—	—	—
Residual urine	−0.006 ± 0.006	0.265	—	—	−0.001 ± 0.006	0.905
Voiding volume	0.001 ± 0.001	0.384	—	—	—	—
Peak flow rate	0.018 ± 0.016	0.248	—	—	0.005 ± 0.017	0.773
MUI	−1.422 ± 0.262	<0.001 *	−1.377 ± 0.264	<0.001 *	−1.391 ± 0.275	<0.001 *
No. of anti-incontinence medications	0.012 ± 0.160	0.942	—	—	0.316 ± 0.191	0.101

Abbreviations: OABSS, Overactive Bladder Symptom Score; BMI, body mass index; MUI, mixed urinary incontinence; Parity, number of childbirths (live births); POP-Q, Pelvic Organ Prolapse Quantification; SE, standard error. ΔOABSS is defined as the difference between OABSS at 3 months and baseline (preoperative) OABSS. Model 1was adjusted for MUI and baseline OABSS. Model 2 was further adjusted for age, BMI, parity, residual urine, peak flow rate, and number of anti-incontinence medications. *: *p* value < 0.05.

**Table 6:** Perioperative and postoperative complications of TVT-O.

Complication	No. of Participants (%)
Bladder perforation	0 (0)
Hematoma/bleeding	0 (0)
Urinary retention	5 (4.5)
Groin or thigh pain	0 (0)
Urinary tract infection	1 (0.9)
Sling exposure/erosion	0 (0)

Abbreviations: TVT-O, transobturator tension-free vaginal tape.

## Data Availability

The data that support the findings of this study are not publicly available because they were obtained from institutional medical records containing de-identified clinical information. Access to these data is restricted by hospital policies and requires approval from the Institutional Review Board.
